# Identification of glycolysis-related clusters and immune cell infiltration in hepatic fibrosis progression using machine learning models and experimental validation

**DOI:** 10.3389/fimmu.2025.1684937

**Published:** 2025-11-05

**Authors:** Guanglin Xiao, Zhiling Deng, Ke Qiu, Aoyi Li, Xingyue Yi, Hong Ren

**Affiliations:** Department of Infectious Diseases, Key Laboratory of Molecular Biology for Infectious Diseases, Ministry of Education, Institute for Viral Hepatitis, The Second Affiliated Hospital, Chongqing Medical University, Chongqing, China

**Keywords:** glycolytic reprogramming, hepatic fibrosis, WGCNA, machine learning, immune cell infiltration

## Abstract

**Objectives:**

Although glycolytic reprogramming constitutes a fundamental driver of hepatic fibrosis (HF), its precise mechanistic contributions remain incompletely characterized. This investigation systematically identified molecular signatures of glycolysis-related genes (GRGs) in HF. We further developed a glycolytic activity-based model for HF risk stratification.

**Methods:**

Integrated analysis of GEO datasets (GSE276114, GSE84044, GSE49541) identified differentially expressed genes (DEGs) associated with HF progression. Integrated weighted gene co-expression network analysis (WGCNA) with six machine learning algorithms to identify core GRGs genes associated with HF progression, and systematically characterized their biological functions and immunoregulatory roles through immune infiltration assessment, functional enrichment, consensus clustering, and single-cell differential state analysis. Glycolytic activity was evaluated in CCl_4_-induced fibrotic mice and TGF-β-stimulated LX-2 cells. Additionally, the expression of core GRGs was validated using immunohistochemical staining and RT-qPCR.

**Results:**

Through the intersection of WGCNA, DEGs, and GRGs, machine learning identified six core GRGs: B3GNT3, CHST4, DCN, GPC3, SOX9, and VCAN. Based on the core GRGs, three GRG-based molecular subtypes were defined. Cluster C, with higher expression of the core GRGs, exhibited significantly enhanced immune infiltration, particularly of adaptive immune cells compared to Cluster A and B. Cluster C comprised a mixed landscape of T cells, mast cells, and pro-fibrogenic cells, distinct from the innate immune-dominant profiles of Clusters A and B. Both *in vivo* and *in vitro* analyses demonstrated enhanced glycolysis in fibrotic progression, accompanied by consistent upregulation of core GRGs.

**Conclusions:**

Glycolytic reprogramming is a key pathogenic driver in HF progression and associated immune infiltration. Investigating this metabolic-immune dysregulation represents a promising therapeutic focus for progression of HF.

## 1 Introduction

Hepatic fibrosis (HF) initially functions as a self-limiting repair mechanism, where transient extracellular matrix deposition preserves tissue integrity during acute injury. This tightly regulated process becomes detrimental when chronic insults (e.g., MASLD) sustain fibrogenic activation, leading to disproportionate scar accumulation that disrupts hepatic architecture and function (1). This maladaptive remodeling drives cirrhosis, which accounts for 2.4% of global mortality and often necessitates liver transplantation due to vascular distortion and regenerative failure (2). Despite decades of research, current antifibrotic strategies remain limited to symptom management rather than targeting core regulators of progressive matrix deposition.

The Warburg effect, originally described in cancer, denotes a metabolic shift from oxidative phosphorylation to aerobic glycolysis. This adaptation allows cancer cells to rapidly generate biomass and energy to support proliferation. Subsequently, this metabolic reprogramming has been observed in fibrotic diseases across multiple organs, including the liver, kidneys, skin, and lungs (3–6). Hepatic stellate cells (HSCs) located in the space of Disse are the primary pro-fibrogenic cells in the liver. Upon activation, the quiescent HSCs transdifferentiate into proliferative, migratory, and contractile myofibroblasts (7). During HSC activation, key glycolytic enzymes, including HK2, PFK1, PKM2, GLUT1, and MCT4, are upregulated, while gluconeogenic enzymes such as PCK1 and FBP1 are downregulated (8, 9). This metabolic shift from oxidative phosphorylation to aerobic glycolysis supports the high energy demands of proliferating HSCs by enabling rapid ATP generation (10).

Glycolysis underpins the functional capacity of innate immune cells (such as inflammatory macrophages, DCs, and neutrophils) and is equally critical for innate-like and adaptive immune cells, particularly in the acquisition of an IL-17+ phenotype (11). Macrophage heterogeneity plays a central role in liver fibrogenesis (12). Resident Kupffer cells, upon activation, initiate and propagate inflammatory responses during liver injury. The transition of macrophages toward an inflammatory phenotype is regulated by metabolic reprogramming of glucose metabolism (13). Inflammatory macrophages engage glycolysis, the pentose phosphate pathway, and a TCA cycle configured for citrate production, alongside nitric oxide generation. Conversely, anti-inflammatory macrophages are dependent on fatty acid oxidation, oxidative phosphorylation, and the arginase pathway (14). In liver fibrosis, this metabolic shift is exploited through PKM2 activation, which augments glycolytic flux and drives macrophage reprogramming toward a profibrogenic phenotype, as demonstrated in mouse models (15).

Glycolysis in HF is under intensive study but far from fully elucidated. HF involves joint action from HSCs, immunocytes, and hepatocytes. However, glycolysis of HF and its impact on the infiltration and activation of immune cells has been scarcely studied. To address this, we combined transcriptomic data from multiple patient cohorts with comprehensive glycolysis-related genes (GRGs) sets to identify core GRGs consistently associated with HF progression. Utilizing machine learning algorithms, we developed a robust diagnostic model and validated its staging performance. Furthermore, we performed consensus clustering to define novel glycolysis-based molecular subtypes and characterized their distinct immunological profiles. Finally, we experimentally validated the relevance of core GRGs and glycolytic phenotypes *in vitro* and in animal models. Our work thus provides a holistic view of glycolytic reprogramming in HF, establishing a foundational resource for future mechanistic and therapeutic exploration.

## 2 Materials and methods

### 2.1 Data sources

Transcriptomic profiles and clinical staging data from patients with HF were obtained from three GEO datasets: GSE276114, GSE84044, and GSE49541. GSE276114 classified 177 specimens into histological fibrosis stages: 39 early fibrosis (F0-F2), 24 severe fibrosis (F3), and 114 cirrhosis (F4) cases (16). GSE84044 provided Scheuer Score-evaluated data encompassing 43 non-fibrotic livers, 20 stage 1, 33 stage 2, 18 stage 3, and 10 stage 4 fibrosis cases (17). GSE49541 contained 72 samples grouped as 40 stage 0–1 versus 32 stage 3–4 fibrosis specimens (18). Based on clinical consensus, stages F0-F2 and F3-F4 were designated mild and advanced fibrosis respectively. Additionally, 315 GRGs were retrieved from the MSigDB. The GSE136103 dataset included single-cell transcriptome data from 5 healthy patient liver tissue samples and 5 cirrhotic patient liver tissue samples (19).

To validate the generalizability of core GRGs in HF, we retrieved two independent GEO datasets: GSE130970 and GSE14323. The GSE130970 cohort comprised 23 healthy controls and 53 fibrosis patients stratified by disease severity (stage 1: n=28, stage 2: n=9, stage 3: n=14, stage 4: n=2) (20). GSE14323 provided transcriptomic profiles from liver specimens of individuals with cirrhosis attributed to hepatitis C virus (HCV) infection or alcohol consumption. Liver tissue samples were classified for the analysis as normal livers (n=19) and HCV cirrhosis (n=41). The characteristics of the samples are described in the **Supplementary Tables 1**, **2** (21). Additionally, gene expression data and corresponding survival information for hepatocellular carcinoma (HCC) cases were acquired from the TCGA.

### 2.2 Data preprocessing and differential gene expression analysis

The efficacy of batch effect correction was validated through two-dimensional principal component analysis (PCA) clustering. Following quality control, we generated a consolidated normalized gene expression dataset using the “limma” package (22). To improve the reliability of differentially expressed genes (DEGs), probe sets for which the adjusted P <0.05, and |log_2_FC| > 0.5 between mild and advanced fibrosis were defined as significant DEGs.

### 2.3 Functional analyses of DEGs and gene set enrichment analysis

Using the “ClusterProfiler” package (23), we conducted comprehensive GO and KEGG enrichment analyses. Statistical significance thresholds were established at a false discovery rate (FDR) < 0.05, with P-values adjusted via the Benjamini-Hochberg procedure for multiple test correction.

### 2.4 Weighted gene co-expression network analysis

WGCNA was implemented to delineate associations between gene modules and pathological traits, enabling identification of hub genes implicated in HF progression. We constructed a similarity matrix using Pearson correlation coefficients, subsequently transformed into adjacency and topological overlap matrices (TOM) through optimized soft-thresholding. Gene clustering was performed via dynamic tree-cutting algorithms with a minimum module size threshold of 50 genes. Modules were designated arbitrary color labels, while module eigengenes (MEs) represented their transcriptional signatures. We identified GRGs critically involved in HF pathogenesis by intersecting these candidate genes with DEGs and pre-defined GRGs.

### 2.5 Development and validation of a diagnostic signature for HF progression using machine learning algorithms

Six distinct machine learning algorithms—Random Forest (RF), Support Vector Machine (SVM), Generalized Linear Model (GLM), Gradient Boosting Machine (GBM), Neural Network (NNET), and Least Absolute Shrinkage and Selection Operator (LASSO)—were employed to assess gene significance. Using the “caret” package, models underwent training with 70% of data (stratified sampling via createDataPartition) and validation on 30% test sets. Model robustness was enhanced through 5-fold repeated cross-validation. Hyperparameter tuning was conducted using the default tuning grids of the caret package, while feature importance ranking was performed through a model-agnostic approach implemented in the “DALEX” package. Core GRGs were derived from intersecting the top-ranked features across all algorithms. Building on this, we established a logistic regression model using the “glm” package. This model subsequently served as the foundation for constructing a clinical nomogram via the “rms” package to stratify HF progression. Performance of the prognostic model was rigorously evaluated through calibration curves, decision curve analysis (DCA), and receiver operating characteristic (ROC) curves.

### 2.6 Gene set enrichment analysis

To elucidate signaling pathway disparities between mild and advanced fibrosis stages, GSEA was implemented (24). Annotated gene sets for disease-relevant pathways were curated from the MsigDB to establish the background reference. Gene sets that were significantly enriched were pinpointed using consistency scores (adjusted p value <0.05).

### 2.7 Gene set variation analysis

Employing an unsupervised, non-parametric methodology, GSVA quantifies pathway-level perturbations by transforming gene-centric expression data into functional enrichment scores (25). This approach characterizes biological activity through comprehensive scoring of predefined gene sets. For the current study, we retrieved pathway-specific gene collections from the MsigDB and computed enrichment profiles using the GSVA algorithm.

### 2.8 Consensus clustering analysis and differences between subtypes

Leveraging expression profiles of hepatic fibrosis-associated GRGs, we performed molecular subtyping of HF patients through unsupervised consensus clustering. This analysis was implemented in R using the “ConsensusClusterPlus” package (26) with the following parameters: 1,000 bootstrap iterations to ensure algorithmic stability, item resampling proportion of 0.8, feature resampling proportion of 1.0, and the k-means clustering algorithm as the foundational method.

### 2.9 Immune cell infiltration analysis

Immune cell infiltration was quantified using both CIBERSORT and ssGSEA. Spearman correlation analysis assessed relationships between immune cell proportions and core GRGs expression, while boxplots visualized infiltration differences of 28 immune cells across GRG-based subtypes.

### 2.10 Analysis of scRNA-seq data

Single-cell RNA sequencing data were processed and analyzed using the “Seurat” package, which facilitated normalization, dimensionality reduction, clustering, and visualization (27). Differentially expressed genes across clusters were identified using the FindAllMarkers function to support cell type classification. Cell identities were determined through a three-step annotation workflow: automated cell type prediction was performed with SingleR, followed by manual verification using well-established marker genes from literature, and finally validated through the CellMarker database (28, 29). Single-cell Phenotype-Associated Subpopulation identifier (scPAS) was applied to quantify the association between individual cells in scRNA-seq data and GRG-based molecular subtypes. Differential expression analysis between high- and low-glycolysis cell subpopulations was performed using the Wilcoxon rank-sum test via the FindAllMarkers function. Genes with log_2_FC > 1 and FDR < 0.05 in high-glycolysis cells were defined as a positive signature, while those with log_2_FC < –1 and FDR < 0.05 in low-glycolysis cells comprised the negative signature. GSVA was then used to compute signature scores for each sample, with a composite score derived by subtracting the negative signature score from the positive signature score (30).

### 2.11 CCl_4_-induced HF model and histological analysis

All experimental procedures involving animals were conducted in compliance with ethical standards approved by the Institutional Animal Care and Use Committee of Chongqing Medical University (Approval No. IACUC-SAHCQMU-2025-0116). To establish fibrosis models, mice received intraperitoneal injections of carbon tetrachloride (CCl_4_) dissolved in corn oil (1:4 v/v) at 1 ml/kg body weight. Administration occurred three times weekly with differential durations: 8 weeks for mild fibrosis induction and 12 weeks for advanced fibrosis induction. Liver sections underwent histological evaluation using standardized Hematoxylin and Eosin (H&E) and Masson’s trichrome staining protocols as described (31).

### 2.12 Immunohistochemistry

Deparaffinized and rehydrated sections underwent antigen retrieval in heated citrate buffer. After peroxidase blocking, tissues were incubated with primary antibody at 4 °C overnight, followed by a secondary antibody and DAB development using a standard IHC protocol.

### 2.13 Cell lines and culture

The human HSC line LX-2 was acquired from Ubigene Biosciences (China). Cells were maintained at 37 °C under 5% CO_2_ atmosphere with saturated humidity. Culture medium consisted of high-glucose DMEM supplemented with 10% FBS (ExCell Bio, China) and 1% penicillin/streptomycin.

### 2.14 EdU staining assay

LX-2 cells were plated in 6-well plates and maintained overnight. The cells were treated with TGF-β (10 ng/ml, Sinobiological) and/or the glycolytic inhibitor 2-DG (2mM, Selleck, USA). EdU incorporation assay was performed with an EdU assay kit (Beyotime, Shanghai, China), according to the manufacturer’s instructions (32). Finally, stain the nuclei with DAPI and observe them using a fluorescence microscope (Nikon, Tokyo, Japan).

### 2.15 Cell migration

Cell migratory capacity was quantified using 8-μm pore Transwell chambers (LABSELECT, China). LX-2 cells were plated in serum-free medium within the upper compartment, while the lower chamber contained 10% FBS. Following 24-hour incubation, traversed cells were fixed with 4% paraformaldehyde and stained with 0.1% crystal violet (Beyotime, China). Invasive cells were manually enumerated across five randomly selected microscopic fields using an inverted fluorescence microscope (Nikon, Japan), with subsequent image acquisition and quantitative analysis.

### 2.16 Lactate and glucose measurement

Lactate concentrations in LX-2 cell supernatant, mice serum, and mice liver tissue were quantified using lactate assay kits (A019-2-1, Nanjing Jiancheng Bioengineering Institute, China), strictly adhering to the manufacturer’s protocol. Glucose concentration in LX-2 cell supernatant was measured using a glucose assay kit (#S0201S, Beyotime, Shanghai, China).

### 2.17 2-NBDG glucose uptake

LX-2 cells were plated in 24-well plates and allowed to adhere overnight. Experimental groups were stimulated with TGF-β (10 ng/ml) for 24 hours. Following treatment, cellular glucose uptake was measured by incubating cells with 0.5 ml 2-NBDG working solution at 37 °C for 60 min per manufacturer’s instructions (Beyotime, China). After removal of supernatant and two PBS washes, nuclei counterstaining was performed. Fluorescence microscopy (Nikon, Japan) was employed to quantify glucose uptake.

### 2.18 Real-time reverse transcription PCR

Total RNA isolation employed Trizol reagent per manufacturer’s protocol (Invitrogen, USA). Subsequent cDNA synthesis utilized a Takara reverse transcription kit (Japan). Primer sequences are detailed in **Supplementary Tables 3**, **4**.

### 2.19 Statistical analysis

The Wilcoxon test was utilized to complete the comparative analysis among the groups. Correlation analyses utilized Spearman’s rank-order method. All analyses were performed in R 4.4.1 and GraphPad Prism 8.0, with P < 0.05 defining statistical significance.

## 3 Results

### 3.1 DEGs between mild and advanced HF

**Figure 1:**
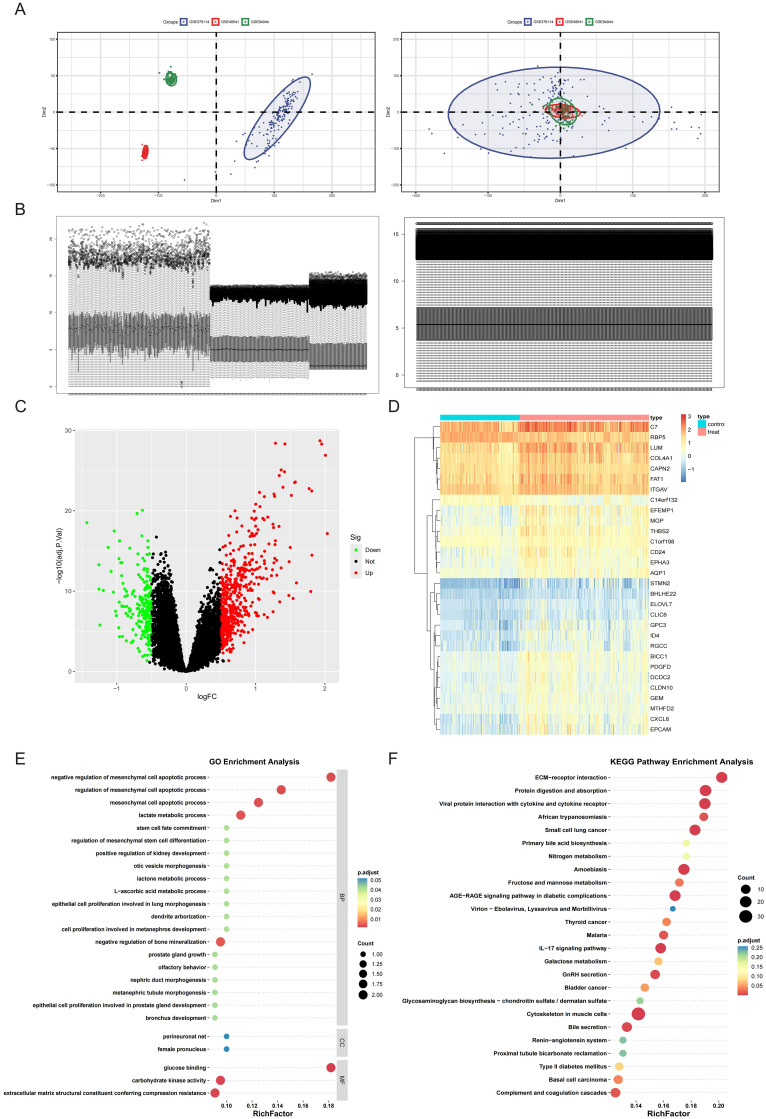
Merging the database and screening differential expressed genes (DEGs). **(A)** Principal Component Analysis (PCA) of samples from the three databases before and after data merging. **(B)** Sample distribution before and after homogenization of the datasets. **(C, D)** DEGs were screened using the limma package under the standard of adj.P.Val<0.05, and |log_2_FC| > 0.5. Upregulated genes were plotted in red and downregulated genes in green in a volcano plot **(C)**. The heatmap **(D)** displays a group of genes differentially expressed in mild and advanced fibrosis. **(E)** GO annotation of the DEGs in association with annotated biological process (BP), cellular component (CC), and molecular function (MF). **(F)** Demonstration of KEGG enrichment results.

To establish a robust integrated cohort, the three HF datasets (GSE276114, GSE84044, and GSE49541) were systematically combined. PCA revealed distinct clustering patterns among fibrotic cases both before and after normalization (**Figure 1A**). Furthermore, distribution characteristics were assessed using box plots, which demonstrated improved comparability after batch adjustment (**Figure 1B**). Comparative profiling revealed distinct transcriptional signatures between mild and advanced HF stages, yielding 517 significantly upregulated and 208 downregulated DEGs. These differential expression patterns were visualized through volcano plot and hierarchical clustering heatmap analyses (**Figures 1C, D**). Subsequent pathway enrichment analysis of hepatic fibrosis-associated DEGs identified critical biological mechanisms. GO analysis demonstrated significant enrichment (P<0.05) in fibrogenic processes, particularly elastic fiber assembly, collagen-activated signaling pathway, and complex of collagen trimers (**Figure 1E**). Complementary KEGG pathway analysis revealed prominent involvement in ECM-receptor interactions, galactose metabolism, and fructose and mannose metabolism (**Figure 1F**). Integrated GO and KEGG analyses indicate that hepatic fibrogenesis is associated with metabolic reprogramming in glycometabolism.

### 3.2 Identification of key genes related to HF progression using WGCNA

**Figure 2:**
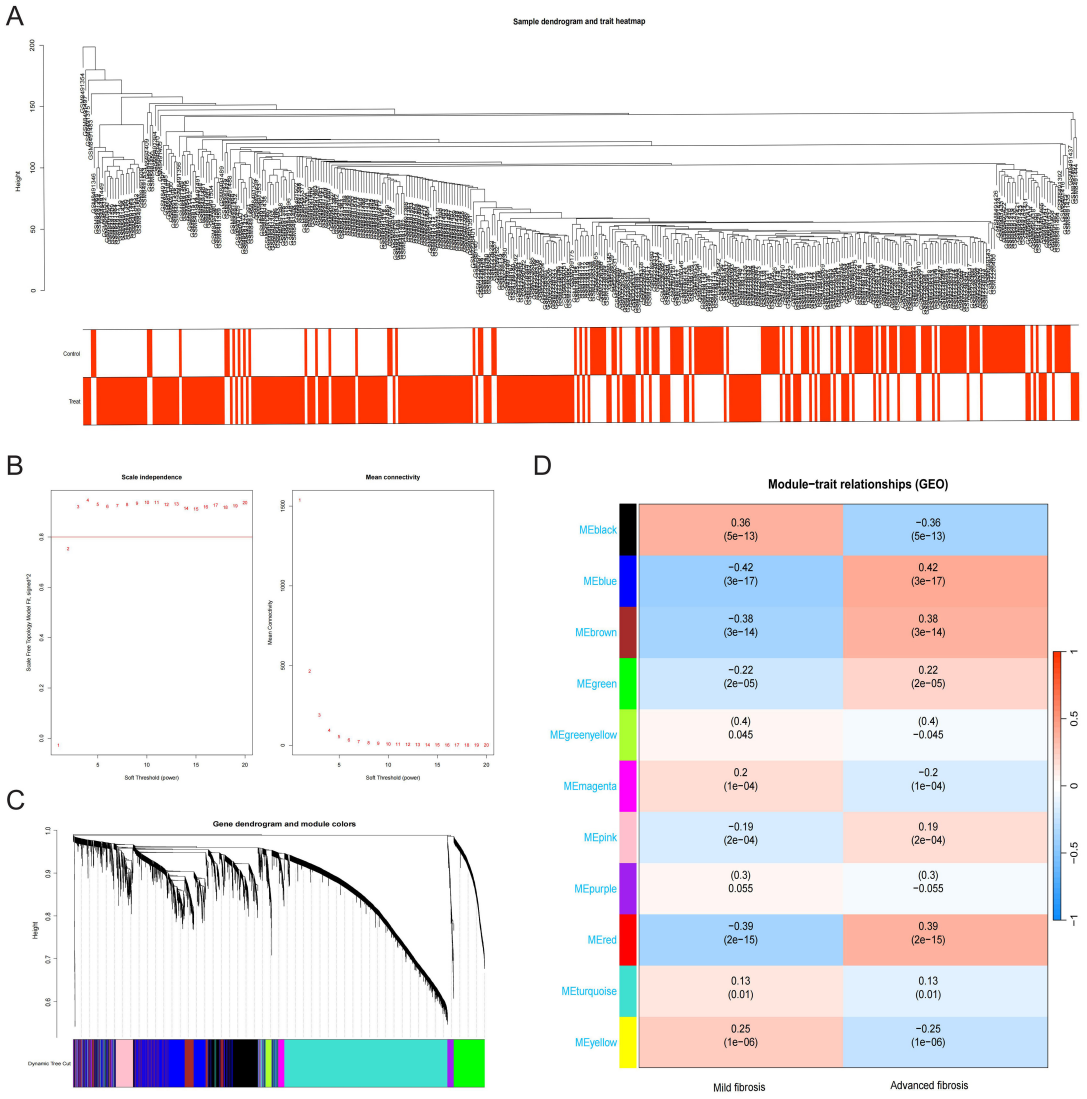
Construction and module analysis of WGCNA. **(A)** Sample dendrogram and trait heatmap. **(B)** Network topology analysis under different soft threshold powers. **(C)** Clustering Dendrogram, illustrating the hierarchical grouping of genes by topological overlap, with the specified module colors representing different gene clusters. **(D)** Correlation analysis for the relationship between different coexpression modules and clinical features.

WGCNA identified HF-associated hub genes through integrated analysis of transcriptomic profiles and clinical metadata. Sample clustering based on Pearson’s correlation coefficients revealed distinct patient subgroups (**Figure 2A**). Optimization of the scale-free topology model determined a soft threshold power of 3, which maximized network interconnectivity while preserving gene similarity (**Figure 2B**). This approach delineated 11 co-expression modules, among which the blue module (976 genes) exhibited the strongest correlation with HF progression (r = 0.42, P = 3 × 10^-17^) (**Figures 2C, D**).

### 3.3 Glycolysis underscores the core predictive DEGs in HF progression

**Figure 3:**
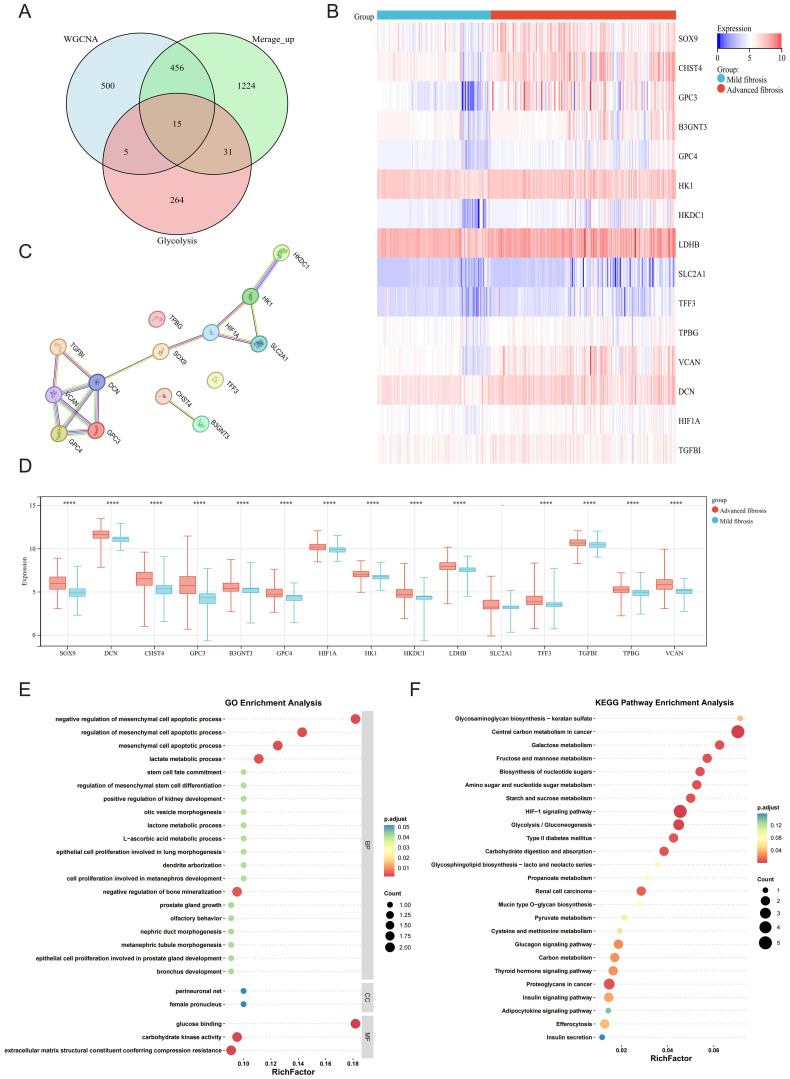
Glycolysis of DEGs in mild and advanced fibrotic livers. **(A)** Venn plot displaying the intersection of upregulated DEGs, WGCNA and glycolysis-related genes (GRGs). **(B)** Heatmap displaying the expression patterns of the 15 GRGs. **(C)** PPI network of 15 overlapping genes. **(D)** Boxplot displaying the expression patterns of the 15 GRGs. **(E, F)** GO annotation and KEGG enrichment analyses of GRGs. *P < 0.05, **P < 0.01, ***P < 0.001, ****P < 0.0001.

Fifteen upregulated GRGs were identified through intersecting three gene sets: 517 upregulated DEGs, 976 key genes from the WGCNA module most relevant to HF progression, and 315 known GRGs (**Figure 3A**). These genes included B3GNT3, CHST4, DCN, GPC3, GPC4, HIF1A, HK1, HKDC1, LDHB, SLC2A1, SOX9, TFF3, TGFBI, TPBG, and VCAN. Heatmap analysis demonstrated pronounced upregulation of these 15 GRGs in advanced fibrosis compared to mild fibrosis (**Figure 3B**). Protein-protein interaction (PPI) network analysis of the 15 GRGs revealed robust connectivity among all genes except TPBG, TFF3, CHST4, and B3GNT3, with the transcription factor SOX9 emerging as a central hub genes (**Figure 3C**). Distinct expression patterns between advanced and mild HF further supported their pathological relevance (**Figure 3D**). Functional enrichment analysis via GO highlighted their association with glycolytic processes (e.g., “lactate metabolic process”) and fibrotic pathways, including “negative regulation of mesenchymal cell apoptotic process” (**Figure 3E**). KEGG pathway analysis underscored their involvement in critical pathways such as “Central carbon metabolism in cancer” and “Glycolysis/Gluconeogenesis” (**Figure 3F**), suggesting a mechanistic link between glycolysis dysregulation and fibrotic progression.

### 3.4 Construction and validation of diagnostic model by machine learning

**Figure 4:**
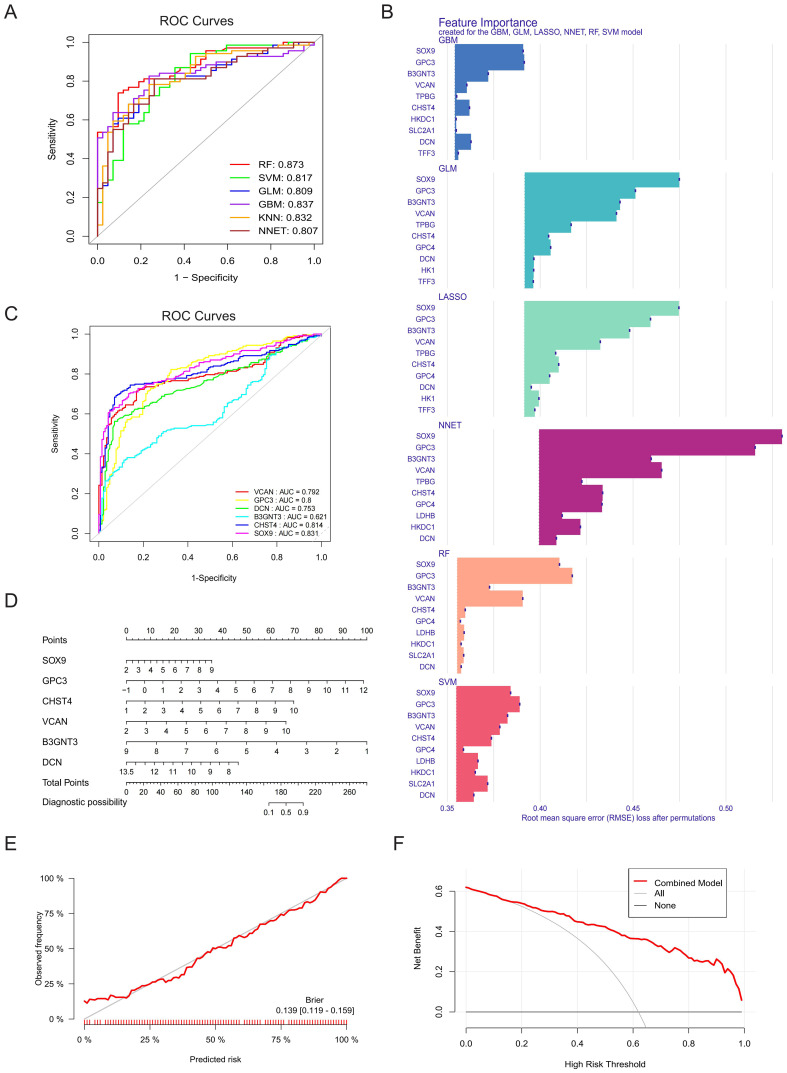
Construction and validation of diagnostic model by machine learning. **(A)** The ROC curves for GBM, RF, GLM, SVM, LASSO and NNET models and their corresponding AUC values. **(B)** The top ten variables in the RMSE ranking used for evaluating the feature importance of the models (GBM, RF, GLM, SVM, LASSO and NNET), and the significance contribution of each model to the input features was analyzed. **(C)** ROC curve and AUC values of core GRGs (B3GNT3, CHST4, DCN, GPC3, SOX9, and VCAN). **(D)** A nomogram of six core GRGs. **(E)** Calibration curve analysis of the prognostic model. **(F)** DCA.

A diagnostic model was constructed using six machine learning algorithms: GBM, RF, GLM, SVM, LASSO, and NNET. Evaluation of the cumulative residual distribution plots and residual boxplots revealed that GBM, RF, GLM, SVM, and LASSO exhibited minimal residuals (**Supplementary Figure S1**), while all six models demonstrated robust discriminative performance for clinical outcomes (AUC > 0.8; **Figure 4A**). The top ten variables for each model, ranked by root-mean-square error (RMSE), were visualized in **Figure 4B**. To identify core diagnostic biomarkers, we extracted the intersection of the top ten most influential genes across all models, yielding six core GRGs: B3GNT3, CHST4, DCN, GPC3, SOX9, and VCAN. ROC analysis confirmed strong diagnostic utility for all genes except B3GNT3 (**Figure 4C**). A nomogram integrating these six core GRGs was subsequently developed using the “rms” package to stratify HF risk (**Figure 4D**). The calibration curve (Brier: 0.139) and DCA collectively demonstrated the model’s robust predictive performance (**Figures 4E, F**).

External validation in the GSE14323 and GSE130970 datasets confirmed the robust discriminative power of these core GRGs. Furthermore, subgroup analyses stratified by etiology (steatohepatitis and viral hepatitis) consistently demonstrated their diagnostic efficacy across distinct fibrotic subtypes (**Supplementary Figure S2**).

### 3.5 Immune landscape analysis across HF progression

**Figure 5:**
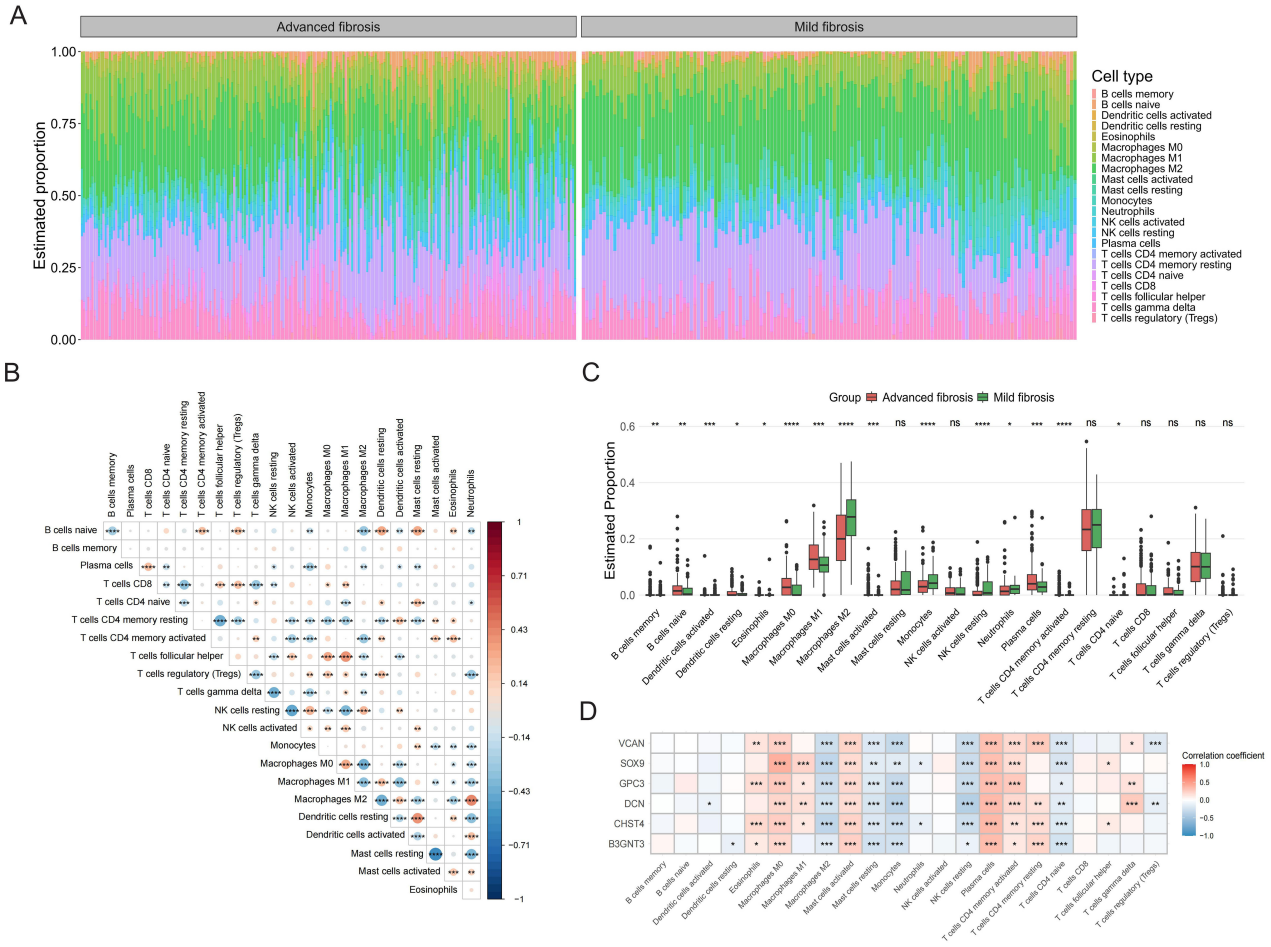
Immune infiltration analysis. **(A)** The bar chart displays immune cell infiltration results of 22 immune cells in each liver fibrosis patients. **(B)** The correlation matrix of immune cell proportions. **(C)** The group comparison chart illustrates differences in the abundance of immune cell infiltration between Cluster C and Cluster A and B. **(D)** Correlation analysis of the core GRGs with immune cells. *P < 0.05, **P < 0.01, ***P < 0.001. ns: no statistically significant (P > 0.05).

Immune infiltration profiling across HF patient samples characterized the relative abundance of 22 immune cell subtypes, visualized via stacked bar chart (**Figure 5A**). In addition, there was a close interplay between immune cells, with a significant negative correlation between M1 macrophages and M2 macrophages, and a strong positive correlation between activated dendritic cells and M2 macrophages (**Figure 5B**). In patients with advanced HF, the proportion of memory B cells, naive B cells, activated mast cells, plasma cells, CD4 memory activated T cells, M0 macrophages, M1 macrophages was found to be higher than those in mild HF. However, the percentage of activated dendritic cells, M2 macrophages, monocytes, resting NK cells, and neutrophils was lower in advanced HF samples (**Figure 5C**). All of the core GRGs had positive correlations with M0 macrophages, activated mast cells, plasma cells and activated T cells CD4 memory and negative correlation with M2 macrophages, resting mast cells, monocytes, resting NK cells and naive T cells CD4 (**Figure 5D**).

### 3.6 GSEA and GSVA analysis of core GRGs

GSEA analysis of six core GRGs revealed distinct pathway enrichments: B3GNT3 was predominantly enriched in integrin αβ-talin-vinculin signaling and translation initiation; CHST4 showed significant enrichment in translation initiation and aberrant TDP-43-mediated electron transfer in complex I; DCN primarily enriched Salmonella SopB-ARNO-ARF-G-actin signaling pathway and ARNO-ARF-G-actin signaling pathway; GPC3 and SOX9 both activated Shigella IpgD-ARNO-ARF-G-actin signaling pathway and ARNO-ARF-G-actin signaling pathway; VCAN was enriched in PI3K signaling pathway and RAS-PI3K signaling pathway (**Supplementary Figure S3**).

GSVA demonstrated that overexpression of all examined genes (B3GNT3, CHST4, DCN, GPC3, SOX9, and VCAN) consistently activated the epithelial-mesenchymal transition (EMT) pathway. Furthermore, angiogenesis was co-activated by CHST4, DCN, GPC3, SOX9, and VCAN, while hypoxia signaling was shared by B3GNT3 and CHST4. Notably, distinct pathways exhibited gene-specific activation: apical junction by B3GNT3, apoptosis by DCN, and TNF-α/NF-κB signaling by the GPC3, SOX9, and VCAN (**Supplementary Figure S4**).

### 3.7 Analysis of core GRGs based on the GSE136103 single-cell dataset

**Figure 6:**
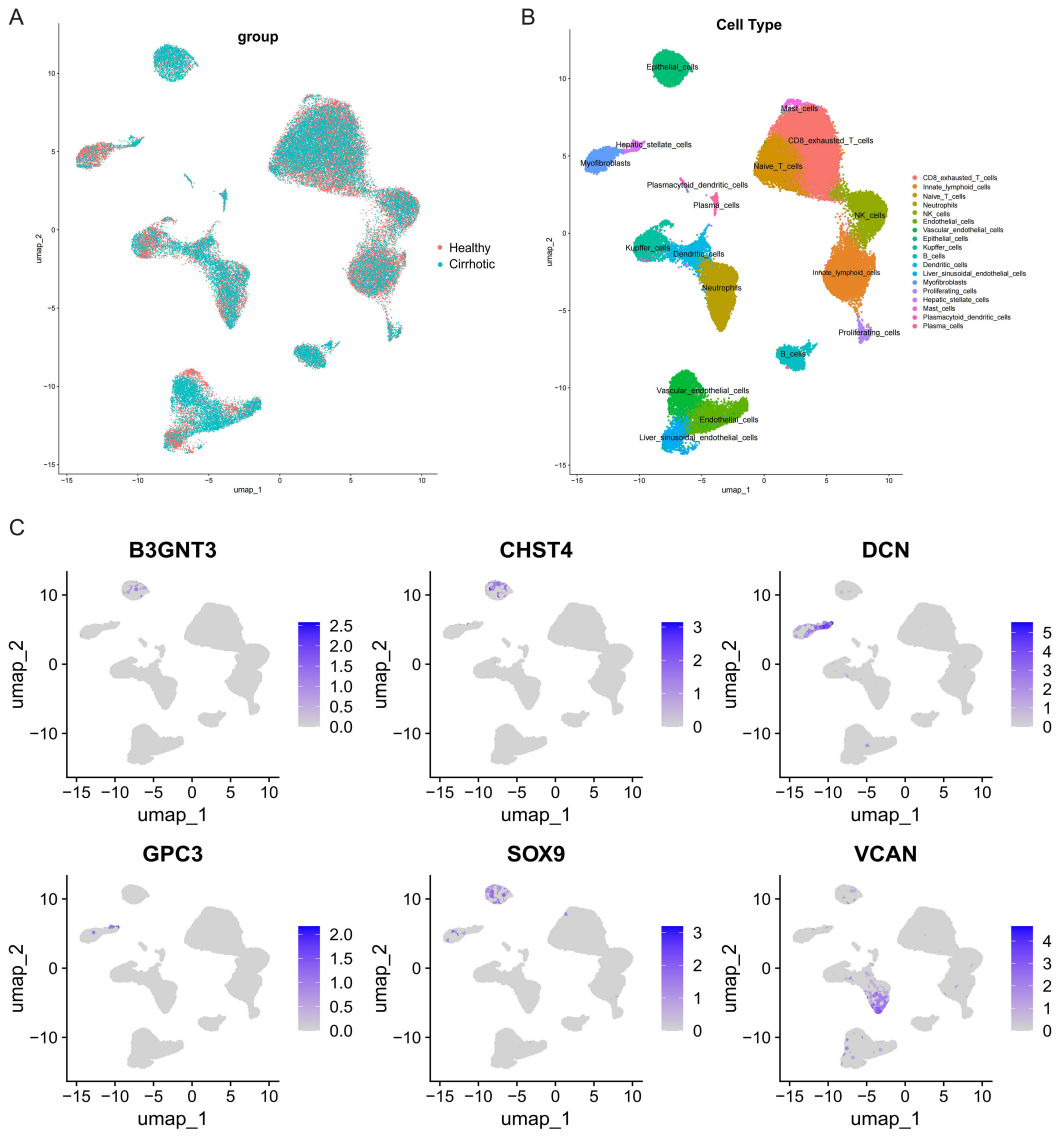
Clustering of GSE136103 scRNA-seq data and identification of cell types. **(A)** UMAP visualization of different group. **(B)** UMAP visualization of different cell types. **(C)** UMAP visualization of core GRGs.

We further explored the expression of the core GRGs in HF patients through scRNA-seq data. After rigorous quality control screening, 51,972 high-quality cells were included in the subsequent analysis. The FindAllMarkers function and the Wilcoxon rank sum test were used to identify specific gene signatures for each cluster. 17 major cell clusters were identified, including exhausted CD8+ T cells (CD3D+CD8A+PDCD1+), Naive/central memory CD8+ T cells (CD3D+CD8A+LEF1+), NK cells (GNLY+NKG7+), B cells (MZB1+CD79A+), plasma cells (MZB1+CD38+), neutrophils (S100A8+VCAN+), conventional dendritic cells (CLEC9A+XCR1+), mast cells (TPSAB1+CPA3+), Liver sinusoidal endothelial cells (LSEC) (CLEC4G+OIT3+), Kupffer cells (ADGRE1+CD68+), endothelial cells(CLEC14A+PLVAP+), HSCs (ACTA2+COL1A1+), innate lymphoid cells (KLRF1+KLRC1+), myofibroblasts (COL1A1+COL3A1+), and vascular endothelial cells (VWF+ACKR1+), epithelial cells (EPCAM+ALB+), proliferating cells (MKI67+), plasmacytoid dendritic cells (LILRA4+LRRC26+) (**Figures 6A, B**). Analysis of the UMAP clustering and expression heatmap showed prominent expression of B3GNT3, CHST4, and SOX9 in epithelial cells; DCN and GPC3 in HSCs and myofibroblasts; and VCAN in neutrophils (**Figure 6C**).

### 3.8 Phenotyping power of the core GRGs in HF

**Figure 7:**
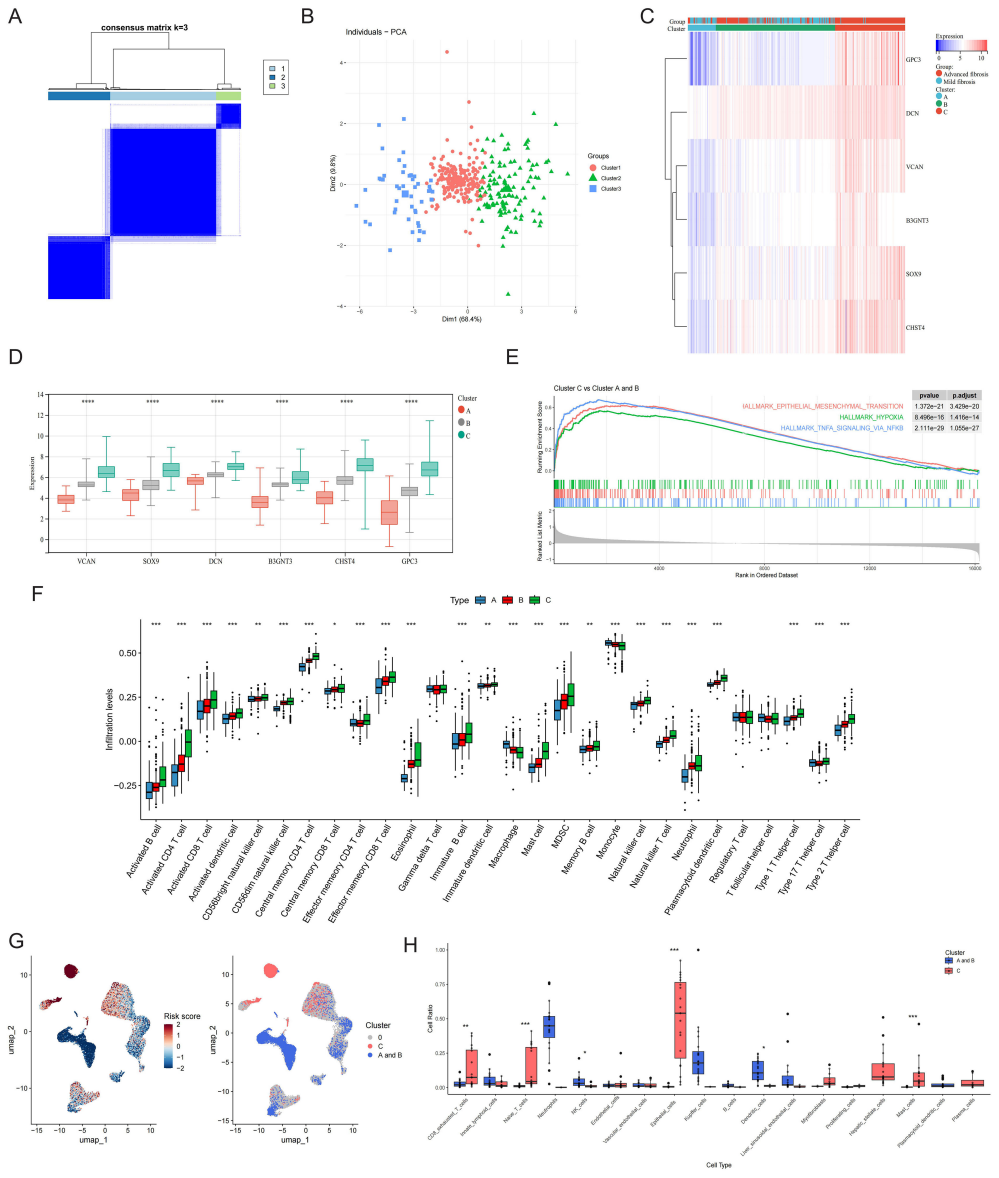
Phenotype clustering by the expression of core GRGs. **(A)** Consensus clustering on liver fibrosis samples based on the six core GRGs. **(B)** PCA of the sample distribution across different phenotypes. **(C)** Heatmap showing the association between gene expression and different phenotypes plotted. **(D)** Expression distinction of core GRGs across different phenotypes. **(E)** GSEA analysis based on the canonical pathways gene sets. **(F)** Box plot showing immune infiltration differences among GRG-based molecular subtypes by ssGSEA. **(G)** The UMAP visualization of the GRG subtype-calculated risk scores and the UMAP visualization of the GRG subtype-selected cells. **(H)** GRG subtype-selected cells with the corresponding bar plots shows the detailed constitutions in each cell type. *P < 0.05, **P < 0.01, ***P < 0.001.

Leveraging core GRG expression profiles, we stratified 373 multi-database samples into three distinct molecular phenotypes (**Figure 7A**). PCA validated clear separation among these subtypes (**Figure 7B**). Clinically, advanced fibrosis cases predominantly accumulated in Clusters C versus Cluster A and B (**Figure 7C**). A gradient expression pattern of core GRGs was observed across the clusters, which correlated with low (Cluster A), intermediate (Cluster B), and high (Cluster C) glycolytic levels (**Figure 7D**). To functionally characterize the differences among GRG-based molecular subtypes identified in our analysis, we performed GSEA comparing the transcriptional profiles of Cluster A and B vs Cluster C. The most notably enriched gene sets in Cluster C were Epithelial-Mesenchymal Transition (EMT), Hypoxia, and TNFA Signaling via NFKB (p < 0.001) (**Figure 7E**). ssGSEA was performed to clarify the characteristics of the three GRG-based molecular subtypes in the immune microenvironment. The proportions of activated B cells, activated CD4 T cells, activated CD8 T cells, central memory CD4 T cells, effector memory CD8 T cells, eosinophils, mast cells, MDSCs, memory B cells, NK cells, NKT cells, plasmacytoid dendritic cells, Th1 cells, and Th2 cells were positively correlated with glycolytic levels. In contrast, the proportions of macrophages and monocytes showed a negative correlation (**Figure 7F**).

Utilizing 373 bulk tissue samples as reference, scPAS analysis identified 3,511 cells associated with Cluster C and 6,791 cells associated with Clusters A and B (**Figure 7G**). Notably, Cluster C was predominantly enriched with immune cell populations, including exhausted CD8+ T cells, naive T cells, mast cells, and plasma cells. Additionally, this cluster contained substantial contributions from epithelial cells, myofibroblasts and HSCs. In contrast, Clusters A and B were primarily composed of NK cells, Kupffer cells, and dendritic cells (**Figure 7H**).

### 3.9 Analysis of the core GRGs in HCC

**Figure 8:**
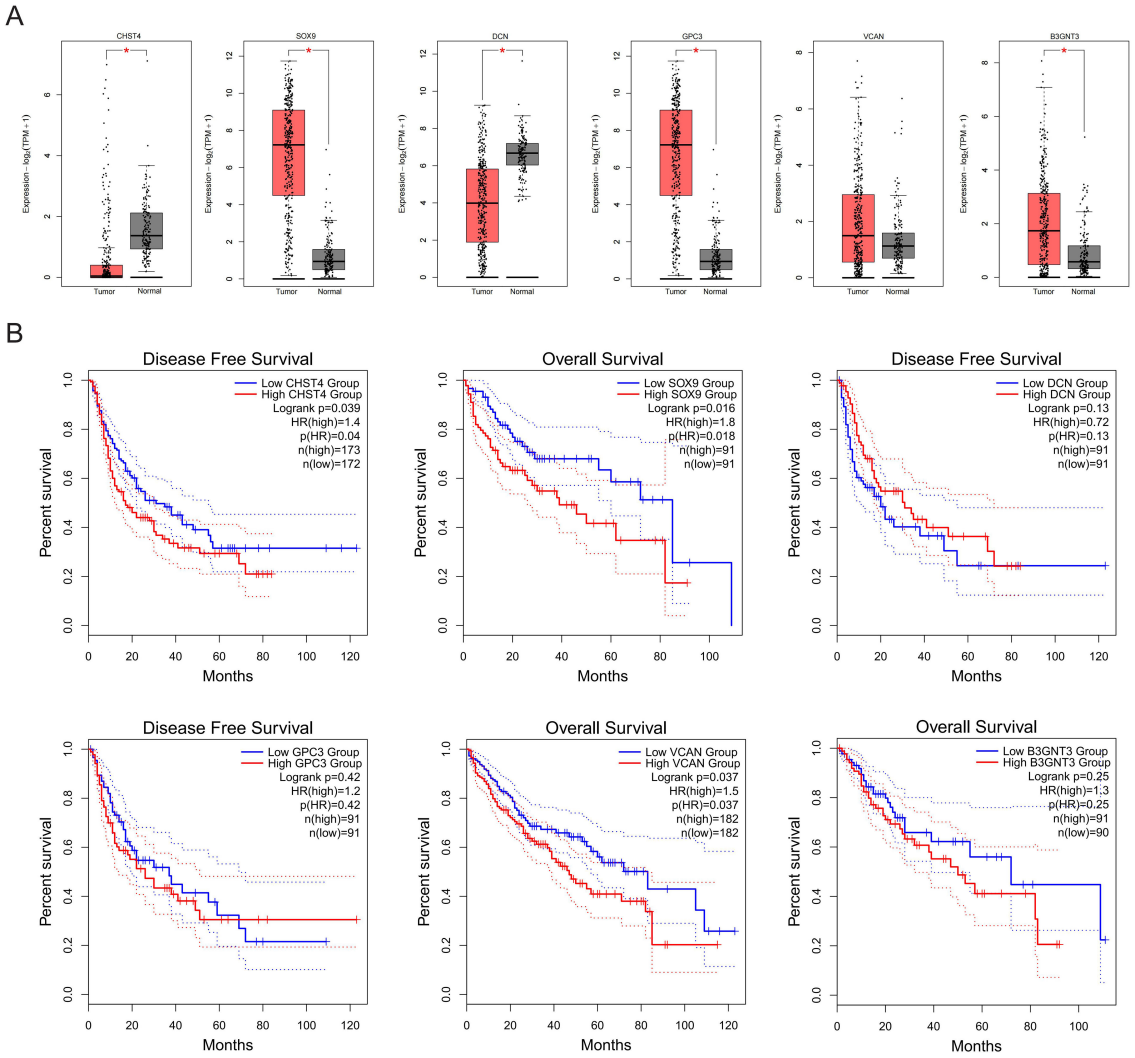
Expression and prognostic values of core GRGs in patients with HCC. **(A)** Expressions of core GRGs in the TCGA-LIHC patients. **(B)** Overall survival of HCC patients with high or low expression of core GRGs.

HF poses significant diagnostic challenges and frequently progresses to malignancy, with many patients presenting at advanced HCC stages. Analysis of TCGA-LIHC data revealed elevated expression of SOX9, DCN, GPC3, and B3GNT3 in tumor tissues versus non-tumor counterparts (**Figure 8A**). Notably, survival analysis identified CHST4 (log-rank P = 0.040), SOX9 (log-rank P = 0.018), and VCAN (log-rank P = 0.037) as prognostic biomarkers significantly correlated with overall survival in HCC patients (**Figure 8B**), implicating these genes as potential oncogenic drivers during fibrotic malignant transformation.

### 3.10 Glycolysis and core GRGs expression CCl_4_-induced HF mice model

**Figure 9:**
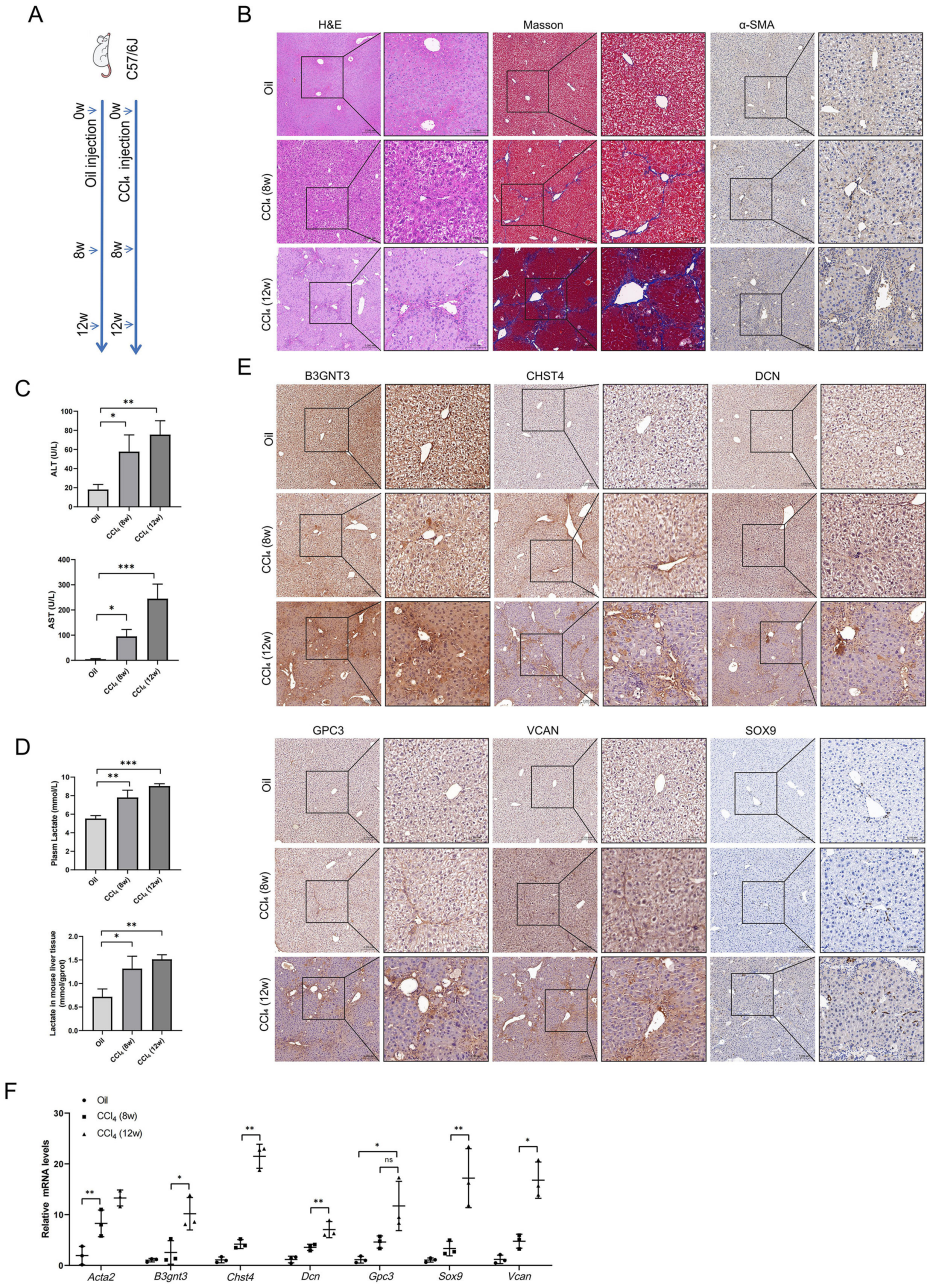
Glycolysis and core GRGs expression in vivo model of liver fibrosis in mice. **(A)** Scheme of protocol. **(B)** Paraffin sections were stained with H&E, Masso’s staining, and anti-α-SMA. **(C)** Plasma ALT and AST levels. **(D)** Lactate levels in plasma and liver tissue. **(E)** Core GRGs were examined by immunohistochemistry. **(F)** Core GRGs were examined by qPCR. Mice, n = 3/group. Data are presented as mean ± SD. *p < 0.05; **p < 0.01; ***p < 0.001.

Mice models of HF with varying severity were induced by intraperitoneal injection of CCl_4_ for 8 or 12 weeks (**Figure 9A**). The results of HE, Masson and IHC staining showed that compared with the oil controls, the CCl_4_ groups had significantly increased collagen deposition (**Figure 9B**). As HF progresses, plasma levels of alanine aminotransferase (ALT) and aspartate aminotransferase (AST) increased (**Figure 9C**). Given that lactate is the end product of glycolysis, we measured lactate levels in both plasma and liver tissues of mice. Our results demonstrated a concomitant elevation in lactate levels with the progression of HF (**Figure 9D**). IHC staining for the core GRGs demonstrated a clear and significant upregulation of these proteins in fibrotic liver tissues compared to oil controls (**Figure 9E**). RT-PCR analyses demonstrated compared to oil controls, CCl_4_-induced fibrotic mice exhibited significant elevation of B3gnt3, Chst4, Dcn, Gpc3, Sox9, and Vcan (all p<0.05) (**Figure 9F**).

### 3.11 Glycolysis and core GRGs expression in LX-2 cells

**Figure 10:**
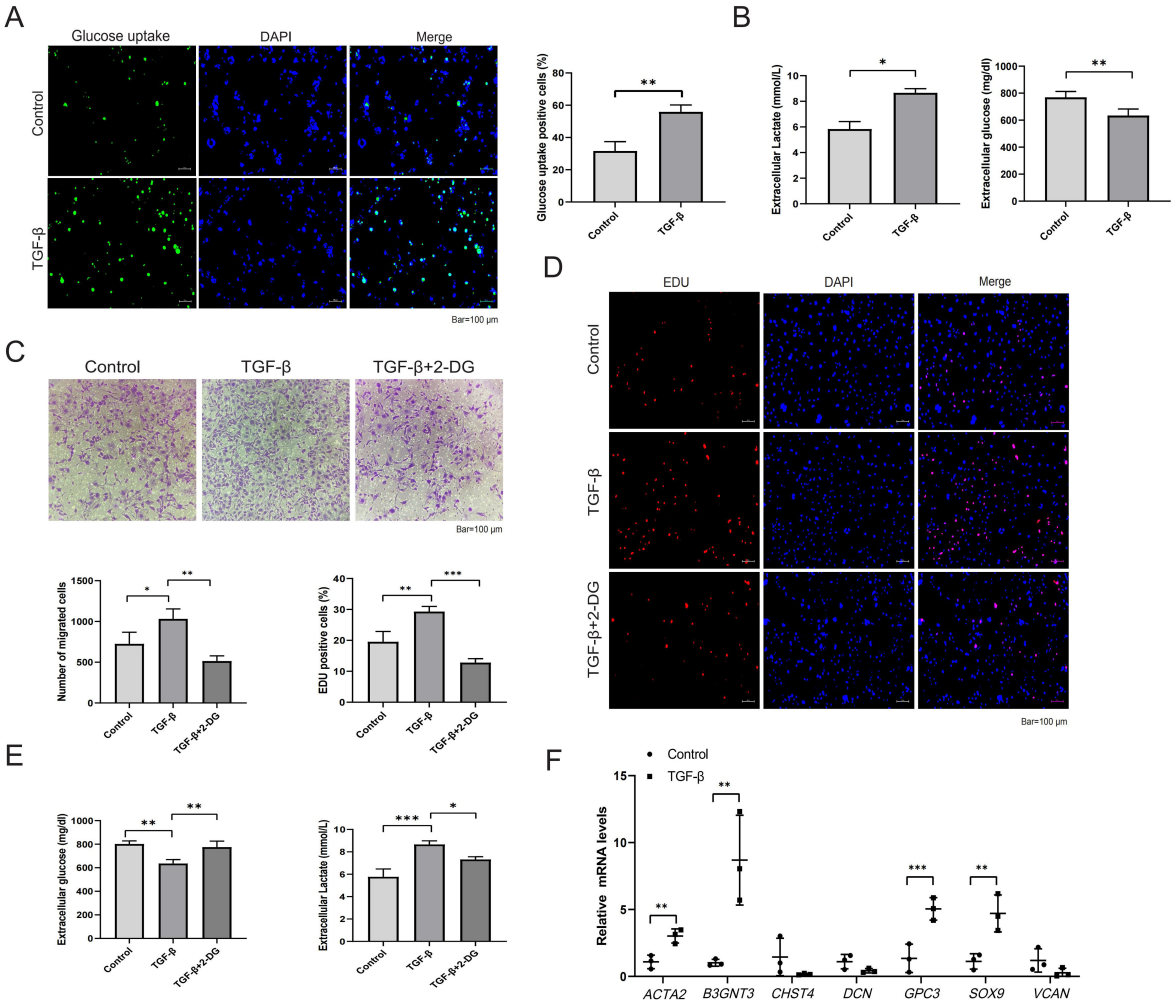
Glycolysis and core GRGs expression in TGF-β-stimulated LX-2 cells. **(A, B)** Glycolytic alterations in LX-2 cells after 48-hour stimulation with TGF-β (10 ng/ml). **(A)** Representative images of glucose uptake with quantifications; **(B)** Glucose consumption and lactate production in LX-2 cell supernatants. **(C-E)** Effects of the glycolytic inhibitor 2-DG on LX-2 cell proliferation, migration, and glycolysis. **(C)** Representative images of transwell migration assay with quantifications; **(D)** Representative images of EdU assay (proliferation) with quantifications; **(E)** Glucose consumption and lactate production; **(F)** Core GRGs were examined by qPCR. n = 3/group. Data are presented as mean ± SD. *p < 0.05; **p < 0.01; ***p < 0.001.

Given that HSC activation is a central event in HF, we further investigated alterations in glycolysis and the expression of core GRGs during LX-2 cell activation. Following TGF-β stimulation, LX-2 cells exhibited increased glucose uptake (**Figure 10A**), reduced supernatant glucose levels, and elevated lactate production (**Figure 10B**). Treatment with the glycolytic inhibitor 2-DG suppressed LX-2 cell migration (**Figure 10C**), proliferation (**Figure 10D**), glucose consumption and lactate production (**Figure 10E**). Finally, we examined the expression of core GRGs (**Figure 10F**). TGF-β stimulation significantly upregulated the expression of B3GNT3,GPC3, and SOX9 (p < 0.05).

## 4 Discussion

This investigation established the prognostic relevance of GRGs in HF progression through integrated analysis of GEO datasets. Initial screening prioritized 15 candidate marker genes (B3GNT3, CHST4, DCN, GPC3, GPC4, HIF1A, HK1, HKDC1, LDHB, SLC2A1, SOX9, TFF3, TGFBI, TPBG, and VCAN). Subsequent validation via six machine learning algorithms consistently identified six core GRGs (B3GNT3, CHST4, DCN, GPC3, SOX9, and VCAN) as robust predictors of HF progression. Immunological landscape analysis further uncovered significant correlations between GRG expression and immune cell infiltration within the fibrotic microenvironment. These findings were experimentally confirmed in a CCl_4_-induced murine HF model, where all six core GRGs demonstrated marked upregulation in fibrotic liver tissues (P<0.05). Collectively, our results delineate a glycolytic regulatory axis driving fibrogenesis and provide mechanistic insights for therapeutic targeting.

Metabolic reprogramming toward enhanced glycolysis represents a pivotal mechanism in fibrogenesis, facilitating fibroblast activation and pathological extracellular matrix deposition (33). Our findings align with prior studies demonstrating SOX9 upregulation in fibrotic tissues, where it promotes myofibroblast differentiation as a key pathological feature of fibrosis (34). Notably, SOX9 modulates disease progression in various pathologies by regulating glycolysis (35, 36). Our findings support this, as we observed a significant SOX9 dysregulation in HF, suggesting a crucial role of glycolysis in the fibrotic process. We additionally identified VCAN as an orchestrator of fibroblast migration/proliferation and collagen deposition (37). DCN, a minor chondroitin-dermatan sulfate proteoglycan in normal liver ECM, becomes progressively upregulated during fibrogenesis and serves as a structural component in cirrhotic ECM scaffolding (38). GPC3 is a heparan sulfate proteoglycan. Although its expression is elevated in HF, numerous studies have demonstrated its utility as a biomarker for HCC (39, 40). The relationship between glycosyltransferase B3GNT3 and carbohydrate sulfotransferase CHST4 in HF remains unreported; however, both enzymes exhibit upregulated expression in cancers (41, 42). Since elevated glycolytic activity is similarly observed in both cancer and HF, the upregulation of these genes in HF may provide a plausible explanation for this shared metabolic alteration.

The distinct immune landscapes associated with glycolysis in liver fibrosis, revealed by bulk and single-cell analyses, reflect complementary biological processes within the chronic inflammatory microenvironment. The broad, positive correlation between glycolytic levels and numerous activated and memory lymphocyte populations in bulk tissue signifies a systemic state of immune engagement. This pattern is characteristic of persistent antigenic stimulation, where adaptive immune cells undergo activation, clonal expansion, and differentiation (43). The concurrent rise in myeloid and innate cells like MDSCs and pDCs further illustrates a coordinated, multi-lineage immune response (44). Previous studies have demonstrated that glycolytic signatures, including LDHA, promote the accumulation and immunosuppressive function of MDSCs. In tumors, glycolytic metabolism orchestrates a molecular network involving the AMPK-ULK1 axis, autophagy, and the transcription factor CEBPB to sustain this MDSC-mediated immunosuppression (45). Single-cell analysis refines this view by identifying the specific metabolic states of key cellular players. The high glycolytic flux in CD8+ exhausted T cells represents a critical adaptation to chronic activation (46). Similarly, the elevated glycolysis in plasma cells is a fundamental requirement for their role as antibody factories, meeting the substantial biosynthetic demands of high-rate protein secretion (47). The convergence of both datasets on mast cells confirms their active participation in the glycolytic milieu, likely fueling their rapid degranulation and cytokine production (48). The finding that naive T cells also display a glycolytic phenotype suggests that the inflammatory microenvironment can impose metabolic reprogramming even on quiescent cells, potentially priming them for future activation (49).

This study has several limitations. First, the functional relationship between glycolytic reprogramming and fibrosis remains primarily correlative in our study. Future work necessitates genetic perturbation of key GRGs in relevant cell models to establish direct causality. Second, although we validated core GRGs in mouse liver, their therapeutic potential remains unexplored. Subsequent studies will employ liver-specific AAV or Cre-loxP systems to modulate these genes in both preventive and therapeutic regimens, with parallel assessment of fibrosis and glycolytic flux. Finally, transcriptome-based immune infiltration algorithms require complementary validation via experimental approaches such as immunohistochemistry and mass cytometry performed directly on liver tissues.

## 5 Conclusions

This study holds significant translational potential. Elevated expression of B3GNT3, CHST4, DCN, GPC3, SOX9, and VCAN may represent therapeutic targets for HF through modulation of glycolytic pathways. The glycolysis-derived model robustly distinguished fibrotic progression stages, enabling early identification of high-risk patients and supporting personalized therapeutic stratification. Collectively, these findings expand the mechanistic understanding of metabolic dysregulation in fibrosis and provide actionable insights for clinical intervention.

## Data Availability

Publicly available datasets were analyzed in this study. This data can be found here: GEO database, https://www.ncbi.nlm.nih.gov/geo/.
