# Supplementary material for: Identification of glycolysis-related clusters and immune cell infiltration in hepatic fibrosis progression using machine learning models and experimental validation
Source: Front Immunol. 2025 Nov 5;16:1684937. doi: 10.3389/fimmu.2025.1684937 (PMC12626809; doi:10.3389/fimmu.2025.1684937)
Supplement: Supplementary file 1 [file SupplementaryFile1.docx]

**Supplementary Online Content**

**Identification of glycolysis-related clusters and immune cell infiltration in hepatic fibrosis progression using machine learning models and experimental validation**

Guanglin Xiao; Zhiling Deng; Ke Qiu; Aoyi Li; Xingyue Yi; Hong Ren^*^

Department of Infectious Diseases, Key Laboratory of Molecular Biology for Infectious Diseases (Ministry of Education), Institute for Viral Hepatitis, the Second Affiliated Hospital, Chongqing Medical University, Chongqing, China.

^*^Corresponding author:

Hong Ren, MD, PhD

1. mail: [renhong0531@cqmu.edu.cn](mailto:renhong0531@cqmu.edu.cn)

**Supplementary Figure 1.** Cumulative residual distribution plots and residual boxplots of the machine learning.

**Supplementary Figure 2.** External validation of core GRGs.

**Supplementary Figure 3.** Gene Set Enrichment Analysis (GSEA) of the six core GRGs in liver fibrosis.

**Supplementary Figure 4.** Gene Set Variation Analysis (GSVA) of six core GRGs in liver fibrosis.

**Supplementary Table 1.** Characteristics of the populations studied (GSE130970).

**Supplementary Table 2.** HCV Cirrhotic tissues (GSE14323).

**Supplementary Table 3.** The mouse primer sequences used in the study.

**Supplementary Table 4.** The human primer sequences used in the study.


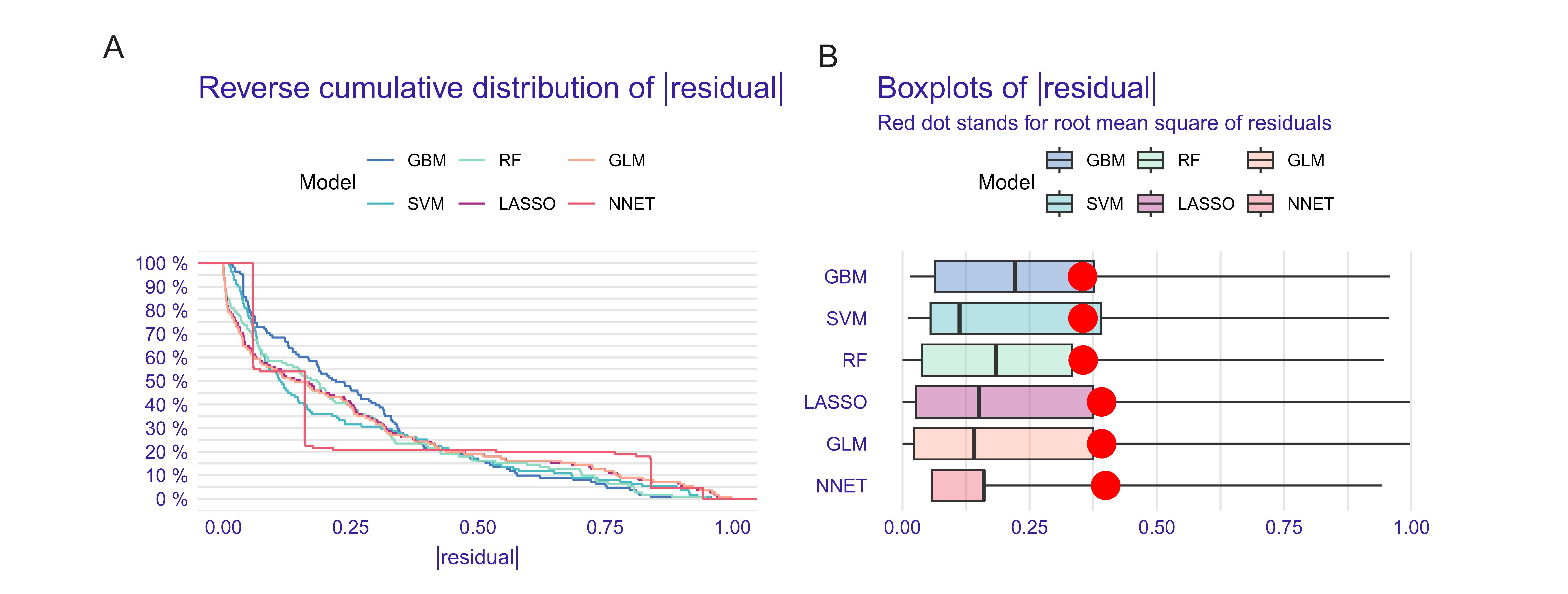


**Supplementary Figure 1. Cumulative residual distribution plots and residual boxplots of the machine learning.** (A) Cumulative residual Distribution: The reverse cumulative distribution of residual for four machine learning models (GBM, RF, GLM, SVM, LASSO and NNET). The curve displays differences in accuracy of the diverse models in fitting the data. (B) Residual box plot: Comparison of the residual distribution of the four models. The red dots represent the residual root-mean-square error (RMSE) of each model.


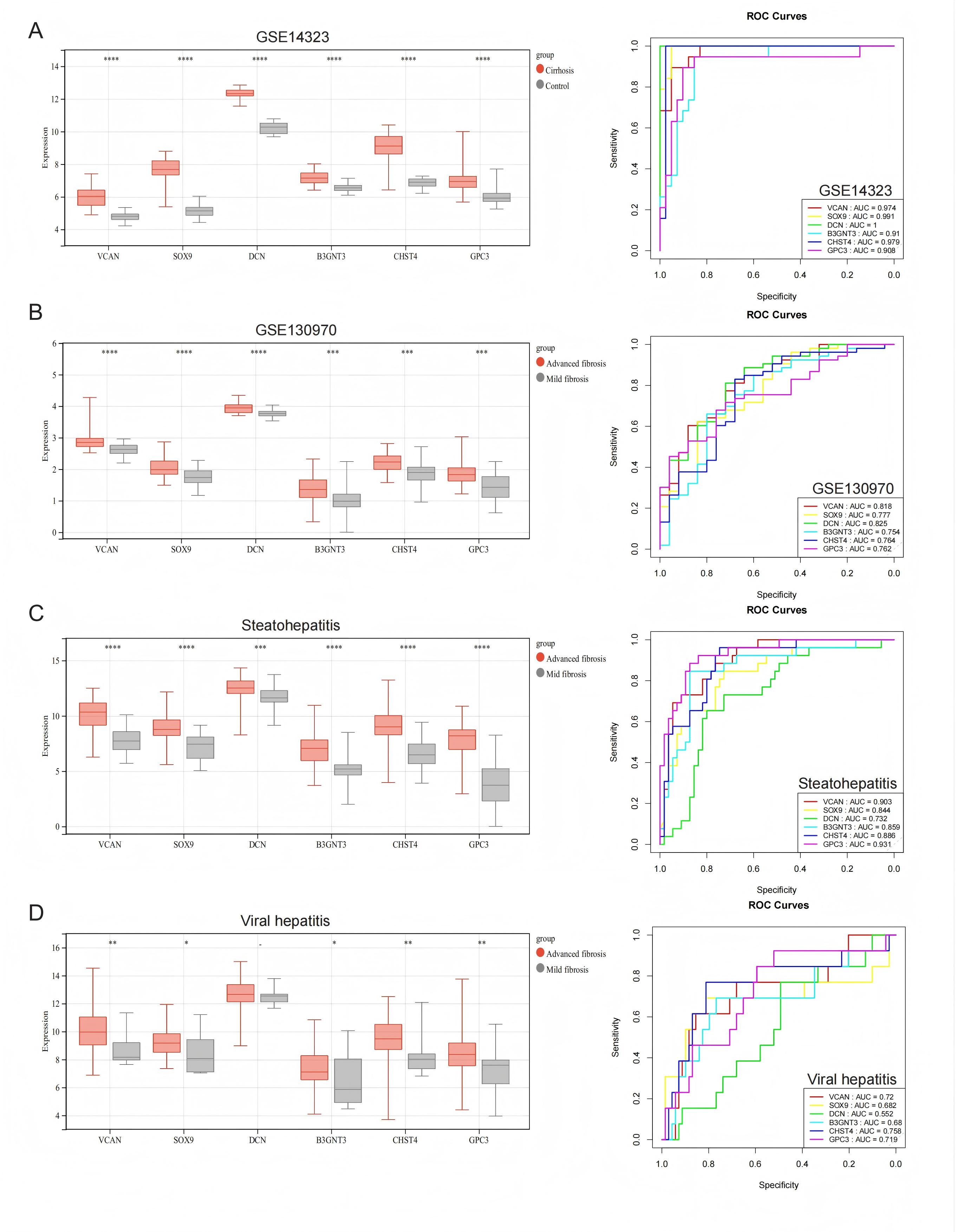


**Supplementary Figure 2. External validation of core GRGs.**


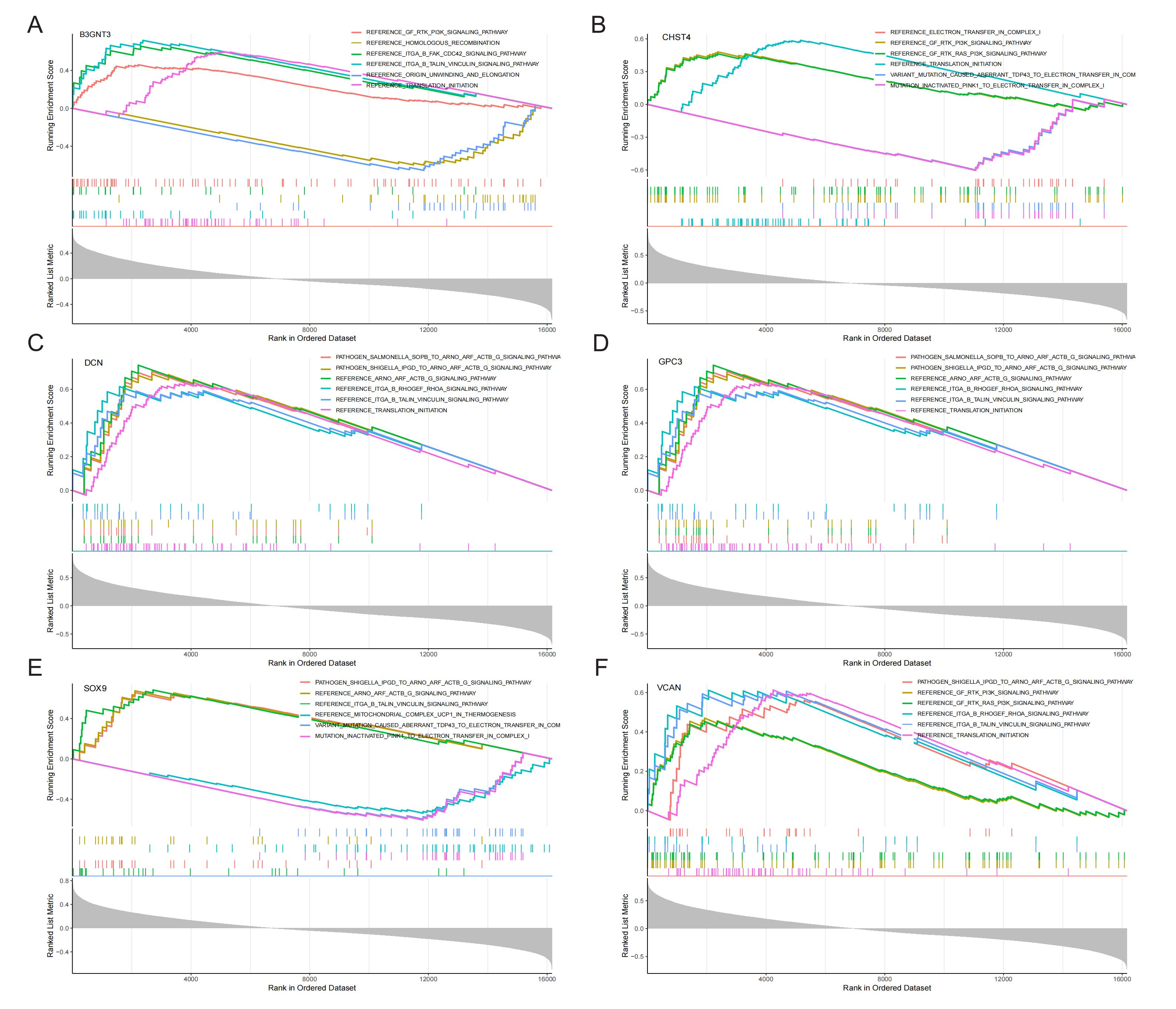


**Supplementary Figure 3.Gene Set Enrichment Analysis (GSEA) of the six core GRGs in liver fibrosis.** (A-F) GSEA results for the six genes: B3GNT3(A), CHST4 (B), DCN (C), GPC3 (D), SOX9 (E), and VCAN (F). Each panel displays the enrichment plots for significantly associated KEGG pathways, ranked by enrichment score.


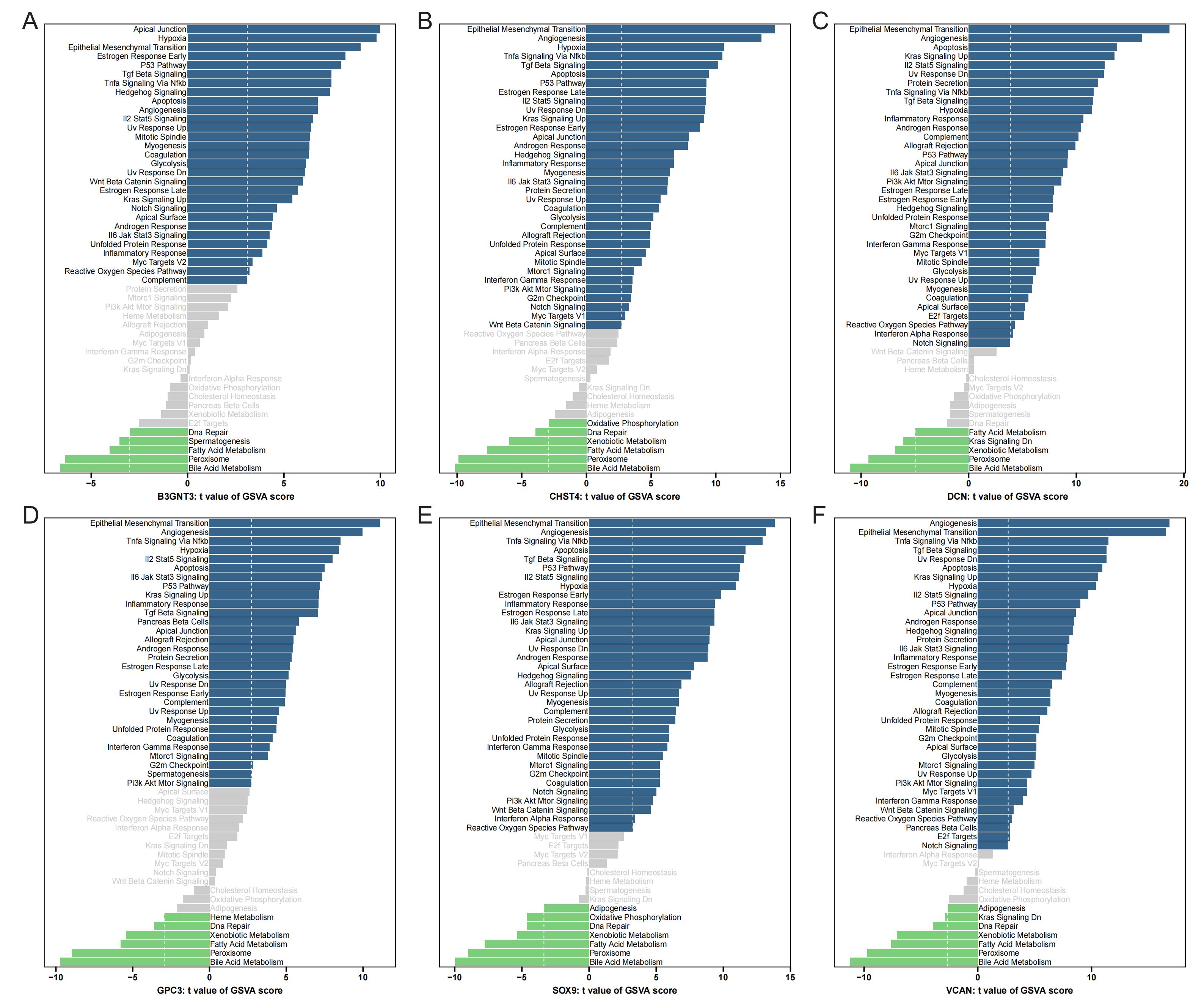


**Supplementary Figure 4. Gene Set Variation Analysis (GSVA) of six core GRGs in liver fibrosis.** (A-F) B3GNT3(A), CHST4 (B), DCN (C), GPC3 (D), SOX9 (E), and VCAN (F). Each plot displays the KEGG pathways significantly associated with each gene, categorized as upregulated (blue), downregulated (green), or not significantly changed (gray). The x-axis shows the t-values of the GSVA scores for each pathway.

| **Supplemental Table 1. Characteristics of the populations studied (GSE130970)** | | |
| --- | --- | --- |
|  | **Histologically normal control (N=6)** | **NAFLD (N=72)** |
| Age (yrs) mean ± S.D. | 49.8 ± 21 | 52.8 ± 12 |
| Gender (males) n | 2 | 28 |
| Caucasians (n) | 5 | 64 |
| Body Mass Index (kg/m2) mean ± S.D. | 25.9 ± 5.8 | 33.3 ± 5.5 |
| Diabetes (n) | 0 | 29 |
| Hypertension (n) | 1 | 46 |
| Hyperlipidemia (n) | 0 | 42 |
| AST (IU/L) mean ± S.D. | 35.7 ± 11 | 60 ± 40 |
| ALT (IU/L) mean ± S.D. | 37.9 ± 12 | 82 ± 56 |
| Alk Phos (IU/L) mean ± S.D. | 96 ± 73 | 93 ± 33 |
| Bilirubin (mg/dl) mean ± S.D. | 0.6 ± 0.2 | 0.6 ± 0.6 |
| Albumin (gm/dl) mean ± S.D. | 4.3 ± 0.2 | 4.4 ± 0.5 |
| Steatohepatitis (n) | 0 | 58 |
| Steatosis grade: mean | - | 2 |
| Lobular inflammation grade: mean | - | 1.4 |
| Hepatocellular Ballooning: mean | - | 1.8 |
| Fibrosis stage: mean | - | 1.3 |
| Distribution of Steatosis grade |  |  |
| 0 | 6 | 2 |
| 1 | - | 29 |
| 2 | - | 27 |
| 3 | - | 14 |
| Distribution of Lobular inflammation |  |  |
| 0 | 6 | 5 |
| 1 | - | 55 |
| 2 | - | 12 |
| Distribution of Hepatocellular ballooning grade |  |  |
| 0 | 6 | 14 |
| 1 | - | 28 |
| 2 | - | 30 |
| Distribution of NAS |  |  |
| 0 | 6 | 0 |
| 1 | - | 2 |
| 2 | - | 9 |
| 3 | - | 18 |
| 4 | - | 17 |
| 5 | - | 18 |
| 6 | - | 8 |
| Distribution of fibrosis stage |  |  |
| 0 | 6 | 19 |
| 1 | - | 28 |
| 2 | - | 9 |
| 3 | - | 14 |
| 4 | - | 2 |

| **Supplemental Table 2. HCV Cirrhotic tissues (GSE14323)** | | | | |
| --- | --- | --- | --- | --- |
| **Sample ID** | **HCV (+)** | **HCC** | **Ishak grade** | **Knodell score** |
| 4-CT | Yes | No | 6 | 15 |
| 6-CT | Yes | No | 6 | 14 |
| 8-CT | Yes | No | 6 | 12 |
| 9-CT | Yes | No | 6 | 13 |
| 10-CT | Yes | No | 6 | 9 |
| 11-CT | Yes | No | 6 | 9 |
| 12-CT | Yes | No | 6 | 10 |
| 13-CT | Yes | No | 6 | 13 |
| 14-CT | Yes | No | 6 | 11 |
| 15-CT | Yes | No | 6 | 10 |
| 16-CT | Yes | No | 6 | 12 |
| 17-CT | Yes | No | 6 | 11 |
| 18-CT | Yes | No | 6 | 13 |
| 21-CT | Yes | No | 6 | 11 |
| 22-CT | Yes | No | 6 | 9 |
| 25-CT | Yes | No | 6 | 13 |
| 27-CT | Yes | No | 6 | 9 |
| 28-CT | Yes | No | 6 | 13 |
| 29-CT | Yes | No | 6 | 11 |
| 31-CT | Yes | No | 6 | 12 |
| 32-CT | Yes | No | 6 | 9 |
| 33-CT | Yes | No | 6 | 12 |
| 34-CT | Yes | No | 6 | 11 |
| 35-CT | Yes | No | 6 | 7 |
| 36-CT | Yes | No | 6 | 7 |
| 37-CT | Yes | No | 6 | 12 |
| 38-CT | Yes | No | 6 | 7 |
| 39-CT | Yes | No | 6 | 11 |
| 40-CT | Yes | No | 6 | 11 |
| 41-CT | Yes | No | 6 | 11 |
| 42-CT | Yes | No | 6 | 9 |
| 43-CT | Yes | No | 6 | 9 |
| 44-CT | Yes | No | 6 | 11 |
| 47-CT | Yes | No | 6 | 14 |
| 47-CT | Yes | No | 6 | 12 |
| 48-CT | Yes | No | 6 | 12 |
| 52-CT | Yes | No | 6 | 11 |
| 54-CT | Yes | No | 6 | 12 |
| 55-CT | Yes | No | 6 | 11 |
| 56-CT | Yes | No | 6 | 11 |
| 57-CT | Yes | No | 6 | 14 |

**Supplementary Table 3. The mouse primer sequences used in the study.**

| **Gene** | **Forward** | **Reverse** |
| --- | --- | --- |
| ACTB | 5’- CACTGTCGAGTCGCGTCC-3’ | 5′- TCATCCATGGCGAACTGGTG -3′ |
| SOX9 | 5′- CAGACCAGTACCCGCATCTG-3′ | 5′- CTCTCGTTCAGCAGCCTCC -3′ |
| DCN | 5′- ACCCGGATTAAAAGGTCGTG -3′ | 5′- AATGGTCCAGCCCAAGAGAC -3′ |
| CHST4 | 5′- GAGCGAGCCTCTCAGTCATT -3′ | 5′- GCGGAAGAAGGAGAAGACCTTAG -3′ |
| GPC3 | 5′- ATCCAGCCGAAGAAGGGAAC -3′ | 5′- TTCTTGTCCGTTCCAGCACA -3′ |
| B3GNT3 | 5′- ATACGGCGACATTCTCCAG -3′ | 5′- AAAGGACCTGCTTAAGCGT -3′ |
| VCAN | 5′- CAAGCCAAAATGGAAACCAG -3′ | 5′- CCCCTTGTAGTCCTGACCAA -3′ |
| ACTA2 | 5′- AGCCATCTTTCATTGGGATGG -3′ | 5′- CCCCTGACAGGACGTTGTTA -3′ |

**Supplementary Table 4. The human primer sequences used in the study.**

| **Gene** | **Forward** | **Reverse** |
| --- | --- | --- |
| ACTB | 5’- CCTTTGCCGATCCGCCG-3’ | 5′-GATATCATCATCCATGGTGAGCTGG -3′ |
| SOX9 | 5′- GGCAAGCTCTGGAGACTTCTG-3′ | 5′- CCCGTTCTTCACCGACTTCC -3′ |
| DCN | 5′- ACATCTCAGCTTTGAGGGCT -3′ | 5′- ATTTTCACAACCAGGGAACCTTT -3′ |
| CHST4 | 5′- CTCAAAAGCAGCAGGGAAGC -3′ | 5′- TGTGCTGAAGTGGAAGACCTTG -3′ |
| GPC3 | 5′- GGGCTAGACTTACAGATTGGCA -3′ | 5′- TTGGCGTTGTTGAGAATGGG -3′ |
| B3GNT3 | 5′- TATGTGTCTGGAGCTTGAGG -3′ | 5′- AAGGATGTGTAGGAGTTCGC -3′ |
| VCAN | 5′- CAAGCCAAAATGGAAACCAG -3′ | 5′- CCCCTTGTAGTCCTGACCAA -3′ |
| ACTA2 | 5′- CTATGCCTCTGGACGCACAACT -3′ | 5′- CAGATCCAGACGCATGATGGCA -3′ |
